# Multicenter, randomized controlled trial of traditional Japanese medicine, kakkonto with shosaikotokakikyosekko, for mild and moderate coronavirus disease patients

**DOI:** 10.3389/fphar.2022.1008946

**Published:** 2022-11-09

**Authors:** Shin Takayama, Takao Namiki, Ryutaro Arita, Rie Ono, Akiko Kikuchi, Minoru Ohsawa, Natsumi Saito, Satoko Suzuki, Hajime Nakae, Seiichi Kobayashi, Tetsuhiro Yoshino, Tomoaki Ishigami, Koichiro Tanaka, Kotaro Nochioka, Airi Takagi, Masaru Mimura, Takuhiro Yamaguchi, Tadashi Ishii, Akito Hisanaga, Kazuo Mitani, Takashi Ito

**Affiliations:** ^1^ Department of Education and Support for Regional Medicine (General and Kampo medicine), Tohoku University Hospital, Sendai, Japan; ^2^ Department of Japanese-Oriental (Kampo) Medicine, Graduate School of Medicine, Chiba University, Chiba, Japan; ^3^ Department of Emergency and Critical Care Medicine, Akita University Graduate School of Medicine, Akita, Japan; ^4^ Department of Respiratory Medicine, Japanese Red Cross Ishinomaki Hospital, Ishinomaki, Japan; ^5^ Center for Kampo Medicine, Keio University School of Medicine, Tokyo, Japan; ^6^ Department of Cardiology, Yokohama City University Hospital, Yokohama, Japan; ^7^ Department of Traditional Medicine, Faculty of Medicine, Toho University, Tokyo, Japan; ^8^ Department of Cardiovascular Medicine, Tohoku University Graduate School of Medicine, Sendai, Japan; ^9^ Clinical Research Data Center, Tohoku University Hospital, Sendai, Japan; ^10^ Department of Neuropsychiatry, Keio University School of Medicine, Tokyo, Japan; ^11^ Division of Biostatistics, Tohoku University Graduate School of Medicine, Sendai, Japan; ^12^ Hospital Bando, Bando, Japan; ^13^ Medical Corporation Mitani Family Clinic, Osaka, Japan; ^14^ Akashi Clinic Kanda, Tokyo, Japan

**Keywords:** kakkonto, shosaikotokakikyosekko, Kampo medicines, COVID-19, herbal medicine

## Abstract

The traditional Japanese (Kampo) medicine, kakkonto with shosaikotokakikyosekko, has antiviral and anti-inflammatory effects. In this randomized trial, patients with mild and moderate coronavirus disease (COVID-19) were randomly allocated to the control group receiving conventional treatment for symptom relief such as antipyretics and antitussives or the Kampo group receiving mixed extract granules of kakkonto (2.5 g) and shosaikotokakikyosekko (2.5 g) three times a day for 14 days in addition to conventional treatment. The main outcome was the number of days until total symptom relief. The secondary outcome was the number of days until each symptom’s relief and whether the disease progressed to respiratory failure. We enrolled a total of 161 patients (Kampo group, *n* = 81; control group, *n* = 80). The results from Kaplan–Meier estimates of symptom relief showed that there are no significant differences between the groups. However, covariate-adjusted cumulative incidence of fever relief considering competitive risk showed that the recovery was significantly faster in the Kampo group than in the control group (HR 1.76, 95% CI 1.03–3.01). Additionally, the risk of disease progression to moderate COVID-19 requiring oxygen inhalation was lower in the Kampo group than in the control group (Risk Difference −0.13, 95% CI −0.27–0.01). No significant drug-related side effects were observed. Kakkonto with shosaikotokakikyosekko is effective for fever relief with suppression of disease progression in COVID-19 patients.

**Clinical Trial Registration:** https://jrct.niph.go.jp/en-latest-detail/jRCTs021200020, identifier [jRCTs021200020]

## 1 Introduction

Coronavirus disease (COVID-19) caused by severe acute respiratory syndrome coronavirus 2 (SARS-CoV-2) is a serious life-threatening viral disease affecting millions of people worldwide. As of 1 June 2022, there had been over 500 million cases and over 6 million deaths (The Center for Systems Science and Engineering at Johns Hopkins University, 2022). The clinical manifestations of COVID-19 include fever, chills, cough, dyspnea, fatigue, body aches, headache, loss of taste or smell, sore throat, congestion or runny nose, nausea or vomiting, and diarrhea (US Centers for Disease Control and Prevention, 2022a), and the condition of approximately 20% of patients progresses to respiratory failure (Wu and McGoogan, 2020).

Many clinical trials have been conducted to demonstrate the efficacy and safety of drug repurposing, such as lopinavir/ritonavir, hydroxychloroquine, nafamostat mesilate, azithromycin, steroid inhalation, metformin, ivermectin, and fluvoxamine; however, the efficacy and safety of these drugs for clinical use remain unclear (Furtado et al., 2020; Patel et al., 2021; Bramante et al., 2022; Gupta A. et al., 2022; Clemency et al., 2022; Quinn et al., 2022). The clinical efficacy of anti-SARS-CoV-2 monoclonal antibodies (Gupta T. et al., 2022; O’Brien et al., 2022) and ribonucleoside analogs (Jayk Bernal et al., 2022) has been demonstrated in COVID-19 patients with mild-to-moderate disease, but these clinical applications have limited applicability in patients with risk factors (Ministry of Health, Labor and Welfare, 2022). Although there are several treatment approaches used, there is no established treatment protocol for the disease, and the drugs used have some reported safety and efficacy issues.

Traditional Japanese medicine, Kampo medicine, which traditionally practiced in Japan based on ancient Chinese medicine, has been applied for acute viral infectious disease. In particular, saikatsugekito (SKGT) reconstructed by Sohaku Asada in the 19th century based on the original Shokan un’yo (Shanghan yunyao in Chinese) has been widely used for acute viral infection. It was used for the symptoms in acute viral infectious diseases, such as headache, chill, fever, malaise, arthralgia, thirst, dry nose, nausea, appetite loss, and even dysphoria. SKGT was also used to treat the patients in Spanish flu. Spread more than 100 years ago and recently it was used applies as the mixture of the extract Kampo medicines with kakkonto (KT) and shosaikotokakikyosekko (SSKKS) (Division of Pharmacognosy, Phytochemistry and Narcotics, 2022). KT has proven antiviral effects (Saito et al., 2021) and pro-inflammatory cytokine modulation (Geng et al., 2019; Arita et al., 2020). SSKKS has proven anti-inflammatory effects, lung tissue protective effects, and inflammatory cytokine modulating effects (Ohtake et al., 2000; Arita et al., 2020). Thus, the combined use of KT and SSKKS can alleviate symptoms of the common cold, pharyngitis, bronchitis, and pneumonia, with multifunctional antiviral and anti-inflammatory effects (Arita et al., 2020). In a multicenter, retrospective, observational study showed that early treatment with Kampo medicines including KT and SSKKS may suppress illness worsening risk in COVID-19 cases without steroid use (Takayama et al., 2022).

This study intended to evaluate the clinical effects of two Kampo medicines on alleviating the symptoms and preventing disease progression using a randomized controlled study approach not previously completed. The main hypothesis of this study was that, compared to conventional treatment alone, the combination of the Kampo medicines KT and SSKKS with conventional treatment would significantly improve patients’ symptoms during the first 14 days of treatment for SARS-CoV-2 infection.

## 2 Materials and methods

### 2.1 Protocol and trial information

The study protocol was published in *Trials* on 2 October 2020, with the title “A multicenter, randomized controlled trial by the Integrative Management in Japan for Epidemic Disease (IMJEDI study-RCT) on the use of Kampo medicine, kakkonto with shosaikotokakikyosekko, in mild-to-moderate COVID-19 patients for symptomatic relief and prevention of severe stage: a structured summary of a study protocol for a randomized controlled trial” (Takayama et al., 2020).

#### 2.1.1 Trial status

Protocol version 1.6 as of 3 March 2022.

Start of application 1 June 2020.

Actual date of first enrollment, 22 February 2021.

Last follow-up date, 16 February 2022.

#### 2.1.2 Trial registration

The trial was registered in the Japan Registry of Clinical Trials (jRCT) jRCTs021200020. Registered on 25 August 2020 (https://jrct.niph.go.jp/latest-detail/jRCTs021200020).

#### 2.1.3 Ethical approval

This protocol was approved by the Ministry of Health, Labour and Welfare Certified Clinical Research Review Board of Tohoku University, Sendai, Miyagi, Japan, on 4 August 2020 (certification no. CRB2180001). The authors certify that this trial received ethical approval from the appropriate ethics committee, as described above. Before inclusion in this study, conscious patients were informed of the purpose and clinical procedures required by the study protocol. The investigators in each hospital or clinic explained the purpose, risks, and benefits of study participation. Patients were also informed of their right to withdraw from the study at any time without explanation and without losing their right to future medical care. Patients were free to leave the study protocol at any stage of the study, withdraw their consent, and, consequently, ask for the elimination of their personal data from the database.

#### 2.1.4 Trial design

This was a multicenter, interventional, parallel-group, randomized (1:1 ratio), investigator-sponsored, two-arm study. It was performed in collaboration with seven medical facilities: Tohoku University Hospital, Chiba University Hospital, Keio University Hospital, Akita University Hospital, Toho University Hospital, Yokohama City University Hospital, and Japanese Red Cross Ishinomaki Hospital.

#### 2.1.5 Participants

Patients were recruited from outpatient clinics, isolation facilities, and the seven hospitals. The inclusion criteria were as follows: 1) diagnosis of SARS-CoV-2 infection with positive nasopharyngeal reverse transcription polymerase chain reaction detecting SARS-CoV-2 RNA; 2) COVID-19 clinical stages mild and moderate stage I; 3) symptomatic; 4) ≥20 years of age; 5) male or female; 6) ability to communicate in Japanese; 7) outpatients and inpatients; and 8) provision of informed consent.

The exclusion criteria were as follows: 1) difficulty providing informed consent due to dementia, psychosis, or psychiatric symptoms; 2) allergy to the Kampo or Western medicines used in this study; 3) pregnancy and lactation; 4) inability to follow-up; 5) participation in another clinical trial or interventional study; 6) hypokalemia or use of oral furosemide or steroids; and 7) determined unsuitable for this study by the attending physician.

The following criteria for symptomatic COVID-19 are used to determine staging in Japan (Ministry of Health, Labor and Welfare, 2022): mild stage, oxygen saturation (SpO_2_) ≥96% with cough without dyspnea; moderate stage I, SpO_2_ 93%–96% with dyspnea and pneumonia findings; moderate stage II, SpO_2_ ≤93% and requiring oxygen administration and treatment; and severe stage, requiring intensive care or mechanical ventilation. Thus, the clinical stage of COVID-19 was determined by physicians according to respiratory symptoms, SpO_2_, and the findings of chest radiography or computed tomography (CT).

#### 2.1.6 Randomization

Patients were randomized (1:1 ratio) to each group using the minimization method, with balancing of the arms based on disease stage severity (mild, moderate stage I) and patient age (<65, 65 to <75, or ≥75 years). Computer-generated random numbers were used for minimization.

#### 2.1.7 Blinding

Blinding (masking): Open-label with no blinding.

#### 2.1.8 Intervention and comparator

Patients in the control group received conventional treatment with some combination of antipyretics, antitussives, or expectorants for symptoms that occurred after SARS-CoV-2 infection; acetaminophen 500 mg was used as needed for a fever of 38°C or higher, up to a maximum of three times a day. Dimemorfan phosphate 30 mg/day was used when the cough was numerical rating scale (NRS: ranging from 0 with no symptoms to 10 with severe symptoms) two or higher. If the cough worsened, tulobuterol 2 mg/day was also used. L-Carbocisteine 750 mg/day was used if the sputum was more than NRS 2. Anti-SARS-CoV-2 monoclonal antibodies, antiviral agents for SARS-CoV-2, and steroid drugs were not used for the treatment.

**FIGURE 1:**
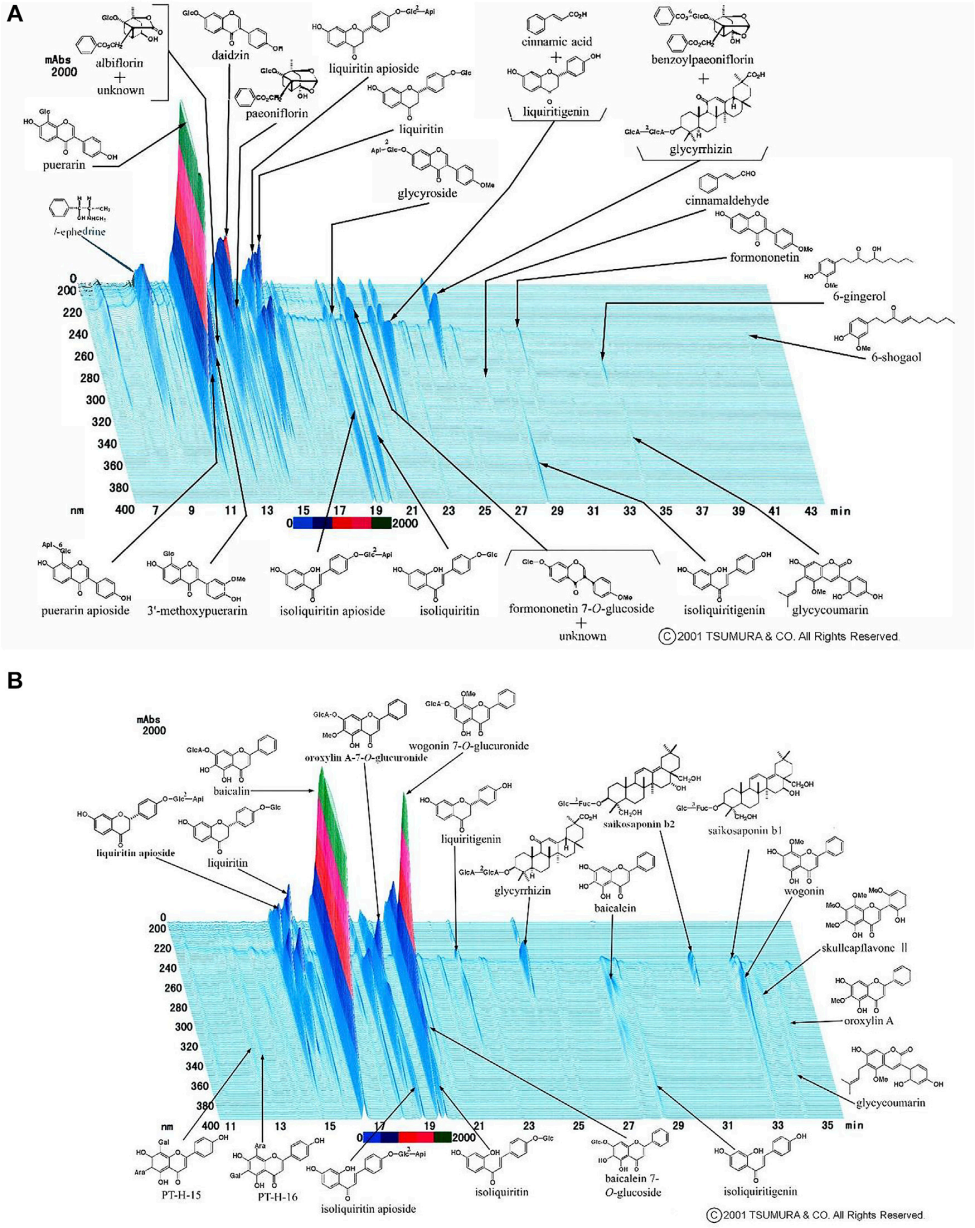
**(A)**High-performance liquid chromatography of kakkonto. **(B)** High-performance liquid chromatography of shosaikotokakikyosekko.

Patients in the Kampo group received mixed extract granules of 2.5 g of KT (@TSUMURA and Co., Tokyo, Japan) and 2.5 g of SSKKS (@TSUMURA and Co., Tokyo, Japan) orally, three times a day, for 14 days in addition to the conventional treatment, as mentioned above. KT extract granules approved for use in Japan contain a dried extract of seven crude drugs: Japanese Pharmacopoeia (JP) Pueraria root, JP Jujube, JP Ephedra herb, JP Glycyrrhiza, JP Cinnamon bark, JP Peony root, and JP Ginger. The results of high-performance liquid chromatography (HPLC) analysis of KT are shown in Figure 1A. SSKKS extract granules contain dried extracts of nine crude drugs: JP Bupleurum root, JP Scutellaria root, JP Ginseng, JP Pinellia tuber, JP Jujube, JP Ginger, JP Glycyrrhiza, JP Platycodon root, and JP Gypsum. The HPLC results for SSKKS are shown in Figure 1B. Detailed information of crude drugs included in KT and SSKKS is shown in Supplementary Tables S1, S2.

### 2.2 Outcomes

#### 2.2.1 Primary outcomes

The primary outcome was the symptomatic relief of at least one common cold-like symptom, such as fever, cough, sputum, fatigue, and shortness of breath, within the first 14 days of treatment. Cough, sputum, fatigue, and shortness of breath were assessed using NRS ranging from 0 (no symptoms) to 10 (severe symptoms). Symptom relief was defined as two-point reduction from the value at the start of treatment on the NRS scale, for at least 2 days in a row. Fever improvement was defined as lowering of body temperature less than 37°C for at least 2 days in a row.

#### 2.2.2 Secondary outcomes

The secondary outcomes were the incidence of progression to severe respiratory failure (at least one of the following features: SpO_2_ ≤93%, respiratory rate ≥30/min, or need for oxygen administration), which was categorized as moderate stage II COVID-19 during the first 14 days of treatment and relief of each symptom (fever, cough, sputum, fatigue, and shortness of breath) in the first 14 days of treatment. Symptom relief was the same definition as Primary endpoint.

#### 2.2.3 Safety outcomes

The incidence of adverse events judged to be unrelated to primary disease was assessed. The number of participants in each group with numbness in the hands and feet, edema, skin rash, other allergic symptoms, and gastric discomfort were also calculated.

### 2.3 Measures

The following history and examinations were completed upon registration: co-morbid diseases, history of vaccination for SARS-CoV-2, body height, body weight, body temperature, blood pressure, pulse rate, SpO_2_, chest X-ray or CT scan of the chest, blood sampling, leukocyte count (Ly), C-reactive protein (CRP), and lactate dehydrogenase (LDH). We also monitored clinical symptoms, such as SpO_2_, fever, and NRS of cough, sputum, fatigue, and shortness of breath.

### 2.4 Sample size

The main research hypothesis of this study was that, compared to conventional treatment alone, the combination of Kampo medicine and conventional treatment would significantly improve patients’ symptoms (i.e., fever, fatigue, cough, sputum, and shortness of breath) during the first 14 days of treatment for SARS-CoV-2 infection. To analyze the primary endpoint, the duration of time before the improvement of at least one symptom (e.g., fever, fatigue, cough, sputum, or shortness of breath) was estimated using the Kaplan-Meier method. Survival curves were compared between groups using the log-rank test. Assuming this method of analysis and based on previous studies reporting the efficacy of Kampo medicine for patients with COVID-19 and H1N1 influenza, the median survival time in the Kampo medicine group was estimated to be 3 days, which was 1.5 times longer in the control group. Assuming a one-sided significance level of 5%, power of 70%, and allocation ratio of 1:1, the required sample size was calculated as 126 cases. To compensate for loss during follow-up and patient exclusion, we planned to include 160 patients in both groups (Kampo group = 80, control group = 80).

### 2.5 Statistical analysis

#### 2.5.1 Primary outcomes

The main research hypothesis of this study was that symptom relief would significantly improve during the first 14 days of treatment in the Kampo group compared to that in the control group. To estimate the duration before the improvement of at least one symptom, survival curves were estimated for each group using the Kaplan-Meier method. Point estimates and confidence intervals for median time to symptom improvement were calculated. Comparisons between groups were conducted using the log-rank test. The Cox regression model was also used to estimate hazard ratios. The significance level was set at 5% on one side. Symptom relief was defined as two-point reduction in common cold-like symptoms (e.g., cough, sputum, fatigue, shortness of breath) on the NRS, compared to the value at the start of treatment, for at least two consecutive days. Fever improvement was defined as lowering of body temperature to <37°C, for at least two consecutive days.

#### 2.5.2 Secondary outcomes

In the incidence of progression to severe respiratory failure, which needed supplemental oxygen during the first 14 days of treatment, if the incidence proportion in the intervention group is significantly lower than that in the control group, the conclusion was that the combination of Kampo medicine prevented disease progression to severe respiratory failure. The groups were compared by calculating the point estimates of the proportion of progression to severe respiratory failure in the first 14 days of treatment for each group, the point estimates of the differences between the groups, and their confidence intervals. Two-tailed test was used with significance set at 5%. Additionally, the time duration before the relief of each symptom (i.e., fever, fatigue, cough, sputum, and shortness of breath) in the first 14 days of treatment was estimated, and between-group comparisons were conducted like those for the primary outcomes. As a supplemental analysis, we compared the two groups in terms of covariate-adjusted cumulative incidence of symptom relief when drop out due to worsening of symptoms was treated as a competing risk. The adjusted covariates included age, severity, duration from onset to enrollment, vaccination, and baseline of each symptom. Baseline was defined as the value of the NRS or the initial body temperature at the start of the treatment. Two-tailed test was used with significance set at 5%.

## 3 Results

### 3.1 Patient characteristics

**FIGURE 2:**
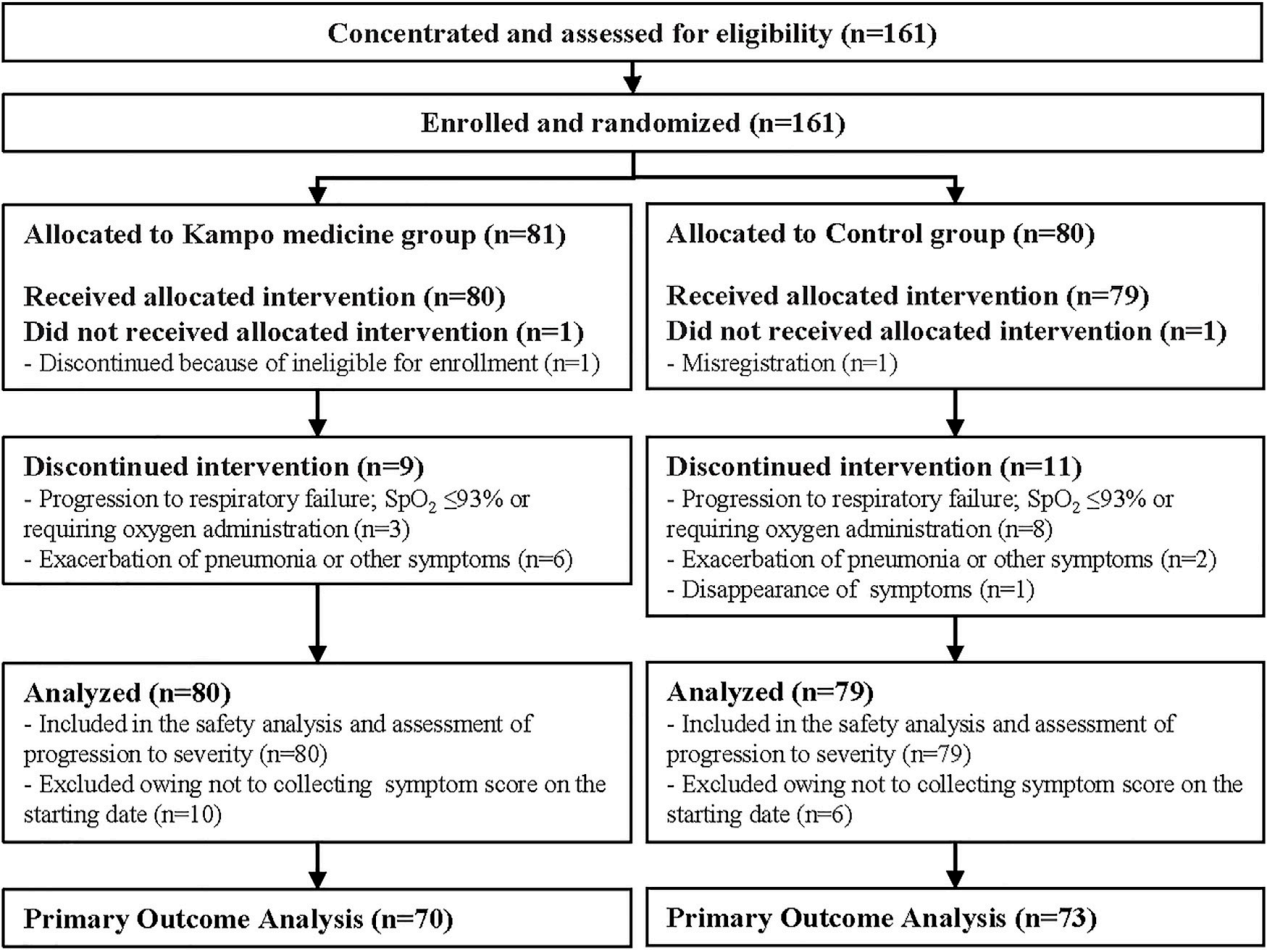
Study flowchart.

A flowchart of the clinical trial is shown in Figure 2. A total of 161 patients were enrolled, and after confirmation of eligibility, they were randomly assigned to the Kampo (*n* = 81) or control (*n* = 80) groups; 80 patients in the Kampo group and 79 patients in the control group received interventions. In total, 70 patients in Kampo group and 73 patients in control group were included in the primary analysis due to their availability to collect analyzable symptom data on the starting date. The demographic characteristics of the patients at baseline are shown in Table 1. Age, sex, risk factors, symptoms, and clinical staging of COVID-19 matched closely in both groups. Regarding age, there were only two patients over 65 years of age. Most enrollment of patients who had been vaccinated coincided with the Omicron variant epidemic period. There were few cases with co-morbidities (such as diabetes mellitus, hypertension, dyslipidemia, cardiovascular disease, respiratory disease, renal dysfunction, and cancer), as shown in Table 1.

**TABLE 1:** Demographic and characteristics of the patients at baseline.

Characteristics	Kampo group (*n* = 70)	Control group (*n* = 73)
Male [n (%)]	45 (64.3)	47 (64.4)
Female [n (%)]	25 (35.7)	26 (35.6)
Age, years [median (IQR)]	35.0 (28.0–47.0)	37.0 (26.0–46.0)
Height, cm [median (IQR)]	166.0 (160.0–173.0)	169.0 (161.5–173.0)
Body weight, kg [median (IQR)]	65.0 (55.5–75.0)	63.0 (56.0–72.0)
Body mass index [median (IQR)]	22.7 (20.7–26.5)	22.6 (20.7–25.6)
Risk factors		
Diabetes mellitus [n (%)]	2 (2.9)	1 (1.4)
Hypertension [n (%)]	6 (8.6)	4 (5.5)
Dyslipidemia [n (%)]	1 (1.4)	2 (2.7)
Cardiovascular disease [n (%)]	1 (1.4)	- (-)
Respiratory disease [n (%)]	2 (2.9)	4 (5.5)
Renal dysfunction [n (%)]	- (-)	- (-)
Cancer [n (%)]	- (-)	- (-)
Smoking habit [n (%)]	22 (31.4)	19 (26.0)
Days from onset to visit, days [median (IQR)]	6.0 (5.0–8.0)	6.0 (5.0–7.0)
Symptoms		
Fever [n (%)]	30 (42.9)	20 (27.4)
Cough [n (%)]	64 (91.4)	65 (89.0)
Sputum [n (%)]	44 (62.9)	51 (69.9)
Fatigue [n (%)]	27 (38.6)	30 (41.1)
Dyspnea [n (%)]	19 (27.1)	22 (30.1)
Chill [n (%)]	1 (1.4)	4 (5.5)
Sweating [n (%)]	1 (1.4)	- (-)
Headache [n (%)]	17 (24.3)	16 (21.9)
Joint pain [n (%)]	- (-)	- (-)
Runny nose [n (%)]	21 (30.0)	18 (24.7)
Nasal obstruction [n (%)]	21 (30.0)	18 (24.7)
Pharyngeal pain [n (%)]	22 (31.4)	16 (21.9)
Thirst [n (%)]	- (-)	- (-)
Chest pain [n (%)]	10 (14.3)	11 (15.1)
Nausea and/or vomiting [n (%)]	2 (2.9)	1 (1.4)
Diarrhea [n (%)]	9 (12.9)	7 (9.6)
Taste disorder [n (%)]	21 (30.0)	18 (24.7)
Smell disorder [n (%)]	25 (35.7)	20 (27.4)
Physical findings		
Body temperature, °C [median (IQR)]	36.60 (36.40–36.90)	36.50 (36.40–36.90)
Pulse rate, beats/min [median (IQR)]	79.0 (70.0–90.0)	80.0 (67.0–90.0)
Systolic blood pressure, mmHg [median (IQR)]	121.0 (112.5–131.0)	126.0 (112.0–134.0)
Diastolic blood pressure, mmHg [median (IQR)]	77.5 (71.0–86.5)	81.0 (74.0–88.0)
SpO_2_, % [median (IQR)]	98.0 (97.0–99.0)	99.0 (98.0–100.0)
Blood sampling test		
WBC count, ×10^3^/μL [median (IQR)]	4.200 (3.260–5.240)	4.220 (3.695–4.945)
Lymphocyte count, ×10^3^/μL [median (IQR)]	1.395 (1.175–1.955)	1.390 (1.100–1.605)
CRP, mg/dL [median (IQR)]	0.600 (0.185–1.400)	0.635 (0.215–1.325)
LDH, U/L [median (IQR)]	188.0 (165.0–238.0)	178.5 (153.5–223.5)
Chest radiography or CT findings; positive [n (%)]	43 (61.4)	41 (56.2)
COVID-19 stage at the first visit		
Mild stage [n (%)]	27 (38.6)	32 (43.8)
Moderate stage I [n (%)]	43 (61.4)	41 (56.2)
Vaccination	7 (10.0)	7 (9.6)
Medication		
Acetaminophen [n (%)]	36 (51.4)	40 (54.8)
Dimemorfan phosphate [n (%)]	64 (91.4)	67 (91.8)
Fexofenadine hydrochloride [n (%)]	7 (10.0)	10 (13.7)
L-Carbocisteine [n (%)]	34 (48.6)	50 (68.5)
Tranexamic acid [n (%)]	6 (8.6)	11 (15.1)
Tulobuterol [n (%)]	10 (14.3)	8 (11.0)

### 3.2 Outcomes

#### 3.2.1 Primary outcomes

**FIGURE 3:**
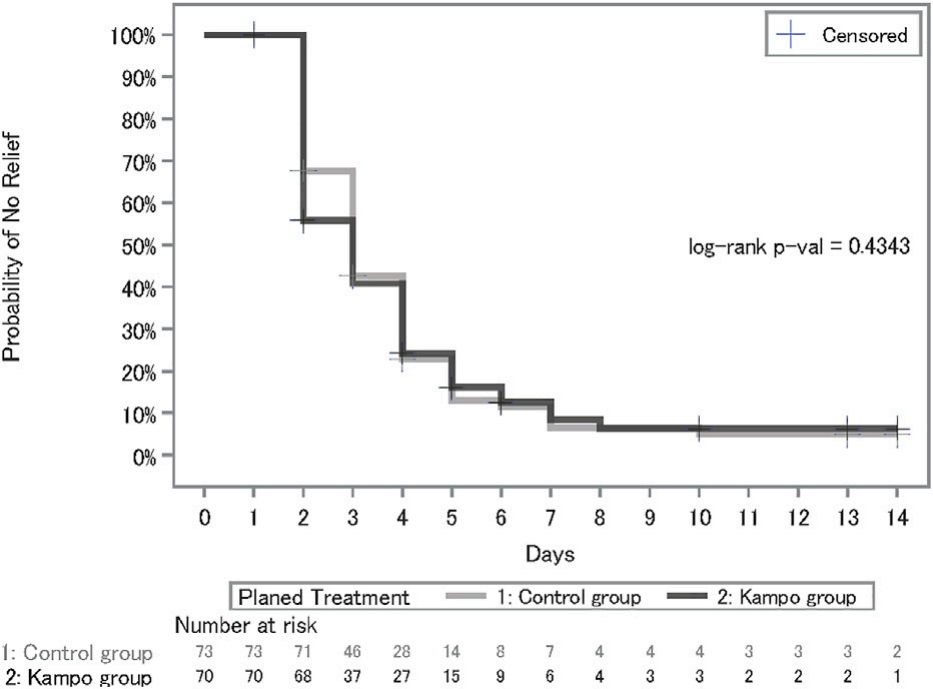
Kaplan–Meier curves of the days till symptomatic relief of at least one of the symptoms, including fever, cough, sputum, fatigue, and shortness of breath.

The Kaplan–Meier estimates for relief of at least one of the symptoms, including fever, cough, sputum, fatigue, and shortness of breath, are shown in Figure 3, and the statistical summary is shown in Table 2. The median number of days until symptomatic relief in the control group (median, 3.0; 90% confidence interval [CI], 3.0–4.0) was similar to that in the Kampo group (median, 3.0; 90% CI, 2.0–4.0) and there were not significant differences between the two groups (*p* = 0.4343 by log-rank test; hazard ratio, 1.02; 90% CI, 0.76–1.38).

**TABLE 2:** Primary and Secondary outcomes.

Outcome	Kampo group	Control group
**At least one symptom**	**(*n* = 70)**	**(*n* = 73)**
Symptom relief [n (%)]	61 (87.1)	64 (87.7)
Median time to Recovery, days [median (90% CI)]	3.0 (2.0–4.0)	3.0 (3.0–4.0)
Hazard Ratio [HR (90% CI)]	1.02 (0.76–1.38)	
**All symptoms**	**(*n* = 7)**	**(*n* = 7)**
Recovery [n (%)]	5 (71.4)	3 (42.9)
Median time to Recovery, days [median (95% CI)]	5.0 (3.0–9.0)	8.0 (3.0–8.0)
Hazard Ratio [HR (95% CI)]	0.88 (0.19–4.00)	
**Fever**	**(*n* = 28)**	**(*n* = 26)**
Recovery [n (%)]	23 (82.1)	18 (69.2)
Median time to Recovery, days [median (95% CI)]	3.0 (2.0–4.0)	4.0 (3.0–5.0)
Hazard Ratio [HR (95% CI)]	1.44 (0.77–2.70)	
**Cough**	**(*n* = 64)**	**(*n* = 65)**
Recovery [n (%)]	50 (78.1)	53 (81.5)
Median time to Recovery, days [median (95% CI)]	5.0 (4.0–6.0)	4.0 (3.0–6.0)
Hazard Ratio [HR (95% CI)]	0.89 (0.60–1.31)	
**Sputum**	**(*n* = 41)**	**(*n* = 45)**
Recovery [n (%)]	32 (78.0)	34 (75.6)
Median time to Recovery, days [median (95% CI)]	6.0 (4.0–7.0)	5.0 (3.0–7.0)
Hazard Ratio [HR (95% CI)]	0.96 (0.59–1.56)	
**Fatigue**	**(*n* = 44)**	**(*n* = 42)**
Recovery [n (%)]	38 (86.4)	35 (83.3)
Median time to Recovery, days [median (95% CI)]	4.0 (3.0–4.0)	3.0 (3.0–5.0)
Hazard Ratio [HR (95% CI)]	1.15 (0.73–1.84)	
**Shortness of breath**	**(*n* = 18)**	**(*n* = 20)**
Recovery [n (%)]	14 (77.8)	15 (75.0)
Median time to Recovery, days [median (95% CI)]	4.0 (3.0–5.0)	4.0 (3.0–6.0)
Hazard Ratio [HR (95% CI)]	1.04 (0.50–2.17)	
**Progression to severe respiratory symptom**	**(*n* = 80)**	**(*n* = 79)**
Number of patients progressed to severe [n (%)]	6 (7.5)	10 (12.7)
Risk Difference [RD (95% CI)]	−0.05 (−0.15–0.04)	

#### 3.2.2 Secondary outcomes

##### 3.2.2.1 Symptom relief

**FIGURE 4:**
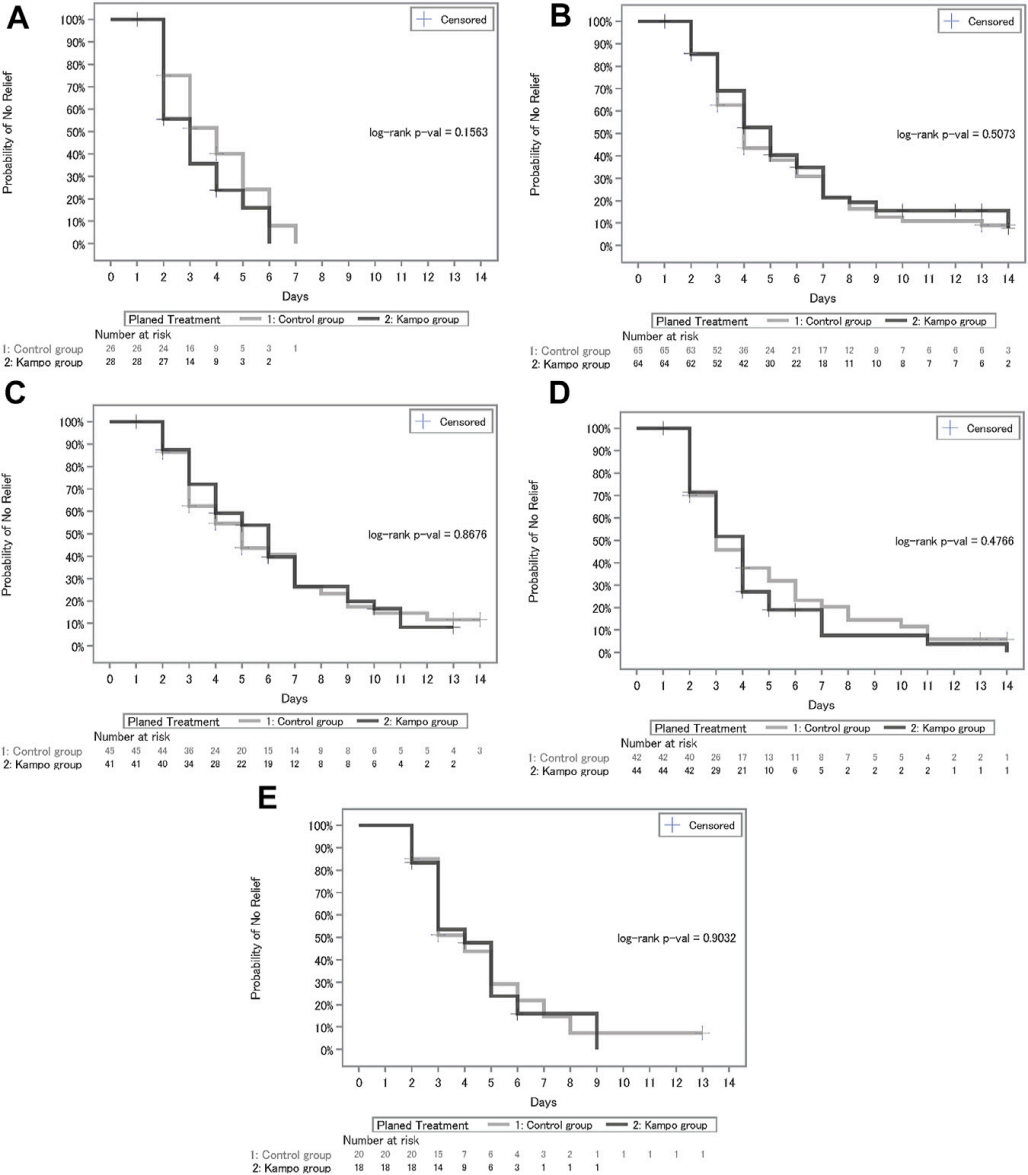
**(A)** Kaplan–Meier curves of days until recovery of fever. **(B)**Kaplan–Meier curves of days until recovery of cough. **(C)** Kaplan–Meier curves of days until recovery of sputum. **(D)** Kaplan–Meier curves of days until recovery of fatigue. **(E)** Kaplan–Meier curves of days until recovery of shortness of breath.

The median number of days until the symptomatic relief and hazard ratio for each outcome, including fever, cough, sputum, fatigue, and shortness of breath, are shown in Table 2 and the Kaplan–Meier estimates are shown in Figure 4. Patients with body temperature at the start of treatment of <37°C were excluded from the analysis population of fever. Regarding cough, sputum, fatigue, and breath shortness, patients with NRS scores at the start of treatment of <2 were excluded from applicable analyses. In the Kampo group, fever decreased relatively faster (median, 3.0; 95% CI, 2.0–4.0) than in the control group (median, 4.0; 95% CI, 3.0–5.0); however, there were no significant differences between the treatment groups (*p* = 0.1563 by log-rank test; hazard ratio, 1.44; 95% CI, 0.77–2.70). There were neither relative nor statistically significant differences between the treatment groups with regards to relief of cough, sputum, fatigue, and shortness of breath as shown in Table 2.

Furthermore, we compared the two groups based on covariate-adjusted cumulative incidence of symptom relief wherein drop out due to primary disease worsening was treated as a competing risk. Age (<45 or ≥45 years), severity (mild or moderate stage I), duration from onset to enrollment (≤7 or >7 days), vaccination, and each baseline symptom were included as adjusted covariates (Table 3). Since the trial unexpectedly included only few elderly patients (aged >65 years), the threshold for age classification was defined as 45 years with reference to higher hospitalization rate in the age class of ≥45 years (Harrs et al., 2021). The threshold for classification of duration from onset to enrollment was defined as 7 days with relation to the high-risk period for disease worsening. The results of covariate-adjusted cumulative incidence analysis showed that recovery of fever was significantly faster in the Kampo group than in the control group (HR, 1.76; 95% CI, 1.03–3.01; *p* = 0.0385), and the relationship between age and duration until fever relief was significant (HR, 2.56; 95% CI, 1.37–4.78; *p* = 0.0031). In regard to relief of cough, sputum, and shortness of breath, there were no significant difference between the treatment groups, whereas the covariates of age and vaccination were significantly associated with duration until symptom relief.

**TABLE 3:** Covariate-adjusted cumulative incidence of symptom relief when drop out due to worsening of symptoms was treated as a competing risk.

Covariates	Hazard ratio (95%CI)	*p*-value
**Fever**		
Treatment: Kampo medicine group vs. Control group	1.76 (1.03–3.01)	0.0385
Age: <45 vs. ≥45, years	2.56 (1.37–4.78)	0.0031
Duration of disease: ≤7 vs. >7, days	0.82 (0.39–1.72)	0.6033
Severity: moderate stage Ⅰ vs. mild	0.95 (0.55–1.65)	0.8651
Vaccination: Yes vs. No	1.77 (0.82–3.82)	0.1434
Baseline of fever, °C	0.57 (0.37–0.90)	0.0148
**Cough**		
Treatment: Kampo medicine group vs. Control group	1.07 (0.76–1.51)	0.7121
Age: <45 vs. ≥45, years	2.30 (1.49–3.56)	0.0002
Duration of disease: ≤7 vs. >7, days	1.21 (0.79–1.87)	0.3779
Severity: moderate stage Ⅰ vs. mild	1.28 (0.86–1.89)	0.2238
Vaccination: Yes vs. No	3.26 (2.29–4.66)	<0.0001
Baseline of cough	1.25 (1.12–1.39)	<0.0001
**Sputum**		
Treatment: Kampo medicine group vs. Control group	1.02 (0.66–1.58)	0.9306
Age: <45 vs. ≥45, year	2.57 (1.52–4.35)	0.0004
Duration of disease: ≤7 vs. >7, days	0.95 (0.55–1.66)	0.8663
Severity: moderate stage Ⅰ vs. mild	1.47 (0.93–2.35)	0.1025
Vaccination: Yes vs. No	3.17 (2.14–4.69)	<0.0001
Baseline of sputum	1.26 (1.06–1.49)	0.0079
**Fatigue**		
Treatment: Kampo medicine group vs. Control group	1.24 (0.83–1.87)	0.2919
Age: <45 vs. ≥45, year	1.52 (0.94–2.47)	0.0905
Duration of disease: ≤7 vs. >7, days	0.89 (0.58–1.37)	0.6092
Severity: moderate stage Ⅰ vs. mild	0.86 (0.55–1.34)	0.4955
Vaccination: Yes vs. No	0.80 (0.19–3.29)	0.7530
Baseline of fatigue	1.03 (0.91–1.16)	0.6547
**Shortness of breath**		
Treatment: Kampo medicine group vs. Control group	1.12 (0.62–2.02)	0.7127
Age: <45 vs. ≥45, year	2.84 (1.17–6.89)	0.0208
Duration of disease: ≤7 vs. >7, days	0.83 (0.45–1.51)	0.5357
Severity: moderate stage Ⅰ vs. mild	1.35 (0.68–2.65)	0.3927
Vaccination: Yes vs. No	3.92 (1.21–12.72)	0.0230
Baseline of shortness of breath	1.04 (0.76–1.44)	0.7912

##### 3.2.2.2 Disease progression to respiratory failure

In all cases, the number of patients that progressed to respiratory failure was 10 and 6 in the control and Kampo groups, respectively, with a smaller risk in the Kampo group than in the control group. However, there was no significant differences between the groups, as shown in Table 2 (risk difference, −0.05; 95% CI, −0.15–0.04; *p* = 0.2786). Patients in the mild stage and who were vaccinated were thought to be less likely to progress to severe respiratory failure because unexpectedly few elderly patients were included in this trial.

Thus, we focused additional assessment only on patients who were unvaccinated and in moderate stage I at the baseline. In this subgroup, the number of patients who progressed to severe respiratory failure in the Kampo group (*n* = 3) was less than half of that in the control group (*n* = 8). This suggests that the Kampo treatment substantially decreased the risk of progression to severe disease (risk difference, −0.13; 95% CI, −0.27–0.01; *p* = 0.0752). None of the patients who progressed to respiratory failure had received a SARS-CoV-2 vaccine.

#### 3.2.3 Safety assessment

##### 3.2.3.1 Adverse events

Adverse events included epigastric disconfort (Kampo group, *n* = 1), gout (Kampo group, *n* = 1), and hand eczema (Kampo group, *n* = 1) (Table 4). No significant differences were observed between the groups.

**TABLE 4:** Adverse events.

Event	Kampo group (*n* = 80)	Control group (*n* = 79)
Any adverse event [n (%)]	3 (3.8)	0 (0.0)
Epigastric discomfort [n (%)]	1 (1.3)	0 (0.0)
Gout [n (%)]	1 (1.3)	0 (0.0)
Hand eczema [n (%)]	1 (1.3)	0 (0.0)

##### 3.2.3.2 Prespecified as events

Numbness in the hands and feet, edema, skin rash and other allergic symptoms, and gastric discomfort were prespecified as events of interest, and the incidence of these events was monitored (Table 5). Their incidence was less in the control group (*n* = 19) than in the Kampo group (*n* = 27); however, there were no significant differences between the groups.

**TABLE 5:** Prespecified as event.

Event	Kampo group (n = 80)	Control group (n = 79)
Any event [n (%)]	27 (33.8)	19 (24.1)
Numbness in the hands and feet [n (%)]	5 (6.3)	1 (1.3)
Edema [n (%)]	5 (6.3)	3 (3.8)
Skin rash and other allergic symptoms [n (%)]	13 (16.3)	10 (12.7)
Gastric discomfort [n (%)]	17 (21.3)	12 (15.2)

## 4 Discussion

The clinical efficacy of Kampo medicine, KT with SSKKS, and conventional treatments was evaluated in a multicenter randomized controlled trial. KT with SSKKS did not significantly shorten the days to symptom relief in patients with mild-to-moderate COVID-19; however, the number of days of fever was shorter in the Kampo group than in the control group. Further, the disease progression in moderate stage I COVID-19 in patients without vaccination for SARS-CoV-2 also tended to be lower in the Kampo group than in the control group. Drug safety was also demonstrated. To the best of our knowledge, this is the first randomized controlled study reporting the efficacy and safety of Kampo medicines for mild-to-moderate COVID-19 patients.

SARS-CoV-2 invades human epithelial cells *via* the angiotensin-converting enzyme 2 (ACE2) receptor and transmembrane protease serine 2 (Hoffmann et al., 2020). After capture in the endosome, the virus is released into the cytoplasm, processed by proteases, and replicated (Jin et al., 2020). In severe cases, the virus also causes cytokine storms (US Centers for Disease Control and Prevention, 2022b). Therefore, multifunctional drugs with both antiviral and immune modulation effects may contribute to symptom relief and suppress disease worsening in COVID-19. Alleviation of the symptoms and improvement of the disease course require multifunctional drugs that have antiviral, anti-inflammatory, and immunomodulatory effects; however, identifying such medications is challenging due to the frequent new variants of the virus.

KT and SSKKS are used for common cold, pharyngitis, bronchitis, and pneumonia (Division of Pharmacognosy, Phytochemistry and Narcotics, 2022). KT is used for the symptoms of acute viral infection and has shown antiviral effects on single-stranded RNA viruses *via* the inhibition of acidification of endosomes (Saito et al., 2021) and pro-inflammatory cytokine modulation, such as interleukin (IL)-1α, IL-6, tumor necrosis factor alpha, interferon gamma, and IL-4 (Geng et al., 2019; Arita et al., 2020) In contrast, SSKKS which is used for the symptoms of subacute viral infection, has shown anti-inflammatory effects, regulating IL-6 levels, lung tissue-protective effects, and inflammatory cytokine-modulating effects (Ohtake et al., 2000; Arita et al., 2020). Thus, the combined use of KT and SSKKS can alleviate symptoms of common cold, pharyngitis, bronchitis, and pneumonia, with multifunctional antiviral and anti-inflammatory effects (Arita et al., 2020). A case series of mild and moderate COVID-19 patients treated with KT and SSKKS has already been reported (Irie et al., 2021a; 2021b). We have reported that early Kampo medicine treatment including KT and SSKKS may suppress illness worsening risk in COVID-19 cases without steroid use in a multicenter, retrospective, observational study (Takayama et al., 2022). The present study was conducted based on these results.

KT and SSKKS included 13 crude drugs: JP Pueraria root, JP Jujube, JP Ephedra herb, JP Glycyrrhiza, JP Cinnamon bark, JP Peony root, JP Ginger, JP Bupleurum root, JP Scutellaria root, JP Ginseng, JP Pinellia tuber, JP Platycodin root, and JP Gypsum. Some crude drugs have demonstrated antiviral effects against SARS-CoV-2. Platycodin D, an ingredient of JP Platycodon root, inhibits both the lysosome- and TMPRSS2-mediated SARS-CoV-2 entry pathways, inhibits exocytosis-mediated membrane fusion, and blocks SARS-CoV-2 entry by preventing cholesterol-dependent membrane fusion (Kim et al., 2021). Ephedrine and pseudoephedrine, which are ingredients of JP Ephedra herb, inhibit the SARS-CoV-2 spike with the ACE2 receptor (Lv et al., 2021). Glycyrrhizin, an ingredient of JP Glycyrrhiza, has been shown to inhibit SARS-CoV-2 replication by inhibiting the main viral protease (van de Sand et al., 2021). Baicalein, an ingredient of JP Scutellaria root, inhibits the 3C-like (3CL) protease in SARS-CoV-2 (Liu et al., 2021). *In silico*, multiple components containing in KT and SSKKS were also identified as candidates to have potential inhibitory activities against SARS-CoV-2. The crude drugs with such potential activities include Bupleurum root, Scutellaria root, Glycyrrhiza (Hejazi et al., 2021; Shakhsi-Niaei et al., 2021; Yu et al., 2021), Ginger (Teng et al., 2021; Zubair et al., 2021.), Pueraria root (Pan et al., 2020), Ephedra herb (Lv et al., 2021), and Cinnamon bark (Prasanth et al., 2021; Husain et al., 2022.). The problem of mutations in the SARS-CoV-2 spike gene have also resulted in the global spread of variants, which decreased vaccine and drug efficacy (Yongbing et al., 2022). Kampo medicines have multifunction with antiviral, anti-inflammatory, and immunomodulatory effects; they may be resistant to viral mutations.

The Kampo medicine maoto includes common pharmacological components with KT, such as ephedrine. Ephedrine has been shown to ameliorate respiratory symptoms by distributing ephedrine in the lung (Matsumoto et al., 2017). Maoto has also been shown to suppress SARS-CoV-2 infection *in vitro* experiments (Kakimoto et al., 2022). Post-exposure prophylaxis with maoto has also shown efficacy in healthcare workers exposed to COVID-19 (Nabeshima et al., 2022). Other Kampo medicines have also been shown to relieve the symptoms of the olfactory disorder (Takayama et al., 2021a; Ono et al., 2022) and refractory chest pain in COVID-19 patients (Arita et al., 2022). Many case reports and case series of patients successfully treated with Kampo medicines have been reported in acute, subacute, and post-COVID-19 conditions (Takayama et al., 2021b).

Several oral antiviral agents have been developed and used in patients with mild and moderate disease. Molnupiravir, a ribonucleoside analog with antiviral activity against RNA viruses, was used to treat COVID-19 with risk factors in a double-blind, randomized, placebo-controlled trial (Jayk Bernal et al., 2022). Early treatment with molnupiravir reduced the risk of hospitalization or death due to risk factors in unvaccinated adults with COVID-19. In a further analysis, molnupiravir showed faster normalization of CRP and SpO_2_, with improvements observed on day 3 of therapy (Johnson et al., 2022). Nirmatrelvir-ritonavir is also used as oral medicine for COVID-19 in patients with risk factors. Nirmatrelvir inhibits the 3CL protease, resulting in antiviral efficacy against SARS-CoV-2. In non-hospitalized COVID-19 patients with a high risk of progression to severe disease, nirmatrelvir-ritonavir reduced the risk of COVID-19-related hospitalization or death. A retrospective cohort study reported the clinical benefits of oral antiviral treatments with molnupiravir or nirmatrelvir-ritonavir in patients not requiring oxygen therapy (Wong et al., 2022). Remdesivir is also a ribonucleoside analog prodrug with antiviral activity against RNA viruses. Early remdesivir infusion for non-hospitalized patients with a high risk of COVID-19 progression led to a lower risk of hospitalization or death than the placebo group in a randomized, double-blind, placebo-controlled trial (Gottlieb et al., 2022). However, in animal studies, molnupiravir has also been shown to be a risk factor for fetal malformations; therefore, contraception use is required for women of reproductive age, and it is contraindicated in pregnant women (Government of Western Australia Department of Health, 2022). Nirmatrelvir-ritonavir includes ritonavir, which requires caution due to the numerous drug interactions (Hammond et al., 2022), and detailed confirmation of medications is required. Remdesivir is inconvenient to use in an outpatient setting because remdesivir is an intravenous formulation. Monoclonal antibody drugs against the SARS-CoV-2, such as a combination of casirivimab and imdevimab and sotrovimab, have been used worldwide. A combination drug of casirivimab and imdevimab (Deeks, 2021) reduced the risk of COVID-19-related hospitalization or death and shortened the median time to resolution of COVID-19 symptoms (Weinrech et al., 2021). Sotrovimab is also a monoclonal antibody directed against the SARS-CoV-2 (Pinto et al., 2020), and it was effective in reducing the risk of hospitalization or death in patients with mild-to-moderate COVID-19 with high risk (Gupta et al., 2022). However, casirivimab or sotrovimab has not shown effective treatment against Omicron BA.2.12.1, BA.4, or BA.5 *in vitro* (Takashita et al., 2022). The safety and efficacy of KT and SSKKS as oral medications for COVID-19 patients are shown in the present study. KT with SSKKS is also inexpensive and already available for use in clinical settings in Japan. Early application for COVID-19 patients may contribute to treating most patients with mild-to-moderate illness who do not require hospitalization by reducing medical expenses and providing economic and medical benefits.

Regarding traditional medicine, a prospective multicenter open-label RCT with a Chinese medicine, the Lianhuaqingwen (LH) capsule, was conducted in COVID-19 patients (Hu et al., 2021). Compared to conventional treatment alone, additional treatment with LH capsules for 14 days significantly increased the symptom recovery rate. However, no significant difference in the conversion rate to the severe stage was observed between the groups. LH is composed of 13 crude drugs, including ephedra herb, glycyrrhiza, and gypsum, which are also commonly included in KT with SSKKS. However, the total components and amounts differed, and the outcome of conversion to the severe stage may differ from that in the present study.

This study has some limitations. First, not all subtypes of SARS-CoV-2 were identified during the registration period, and the influence of subtypes could not be excluded. Second, sample size estimation was performed according to the RCT on H1N1 influenza using Kampo medicines; the sample size may be too small to prove the hypothesis in the present study. Third, it required days for treatment to begin after confirming the positive SARS-CoV-2 Polymerase Chain Reaction test result because delays occurred due to instructions from the municipal health department. The mean number of days before the first visit was six in the present study, which was influenced by this delay, particularly in 2020. Fourth, in Japan, COVID-19 vaccination in elderly individuals began in April 2021 and subsequently, gradually included middle-aged and young adults. There were very few vaccinated patients in the study to make any predictions regarding the use of Kampo medications with vaccinated patients. Finally, this study was an open-label, randomized controlled trial, which cannot exclude placebo bias. To further evidence, double blinded randomized controlled trials should be performed in the future.

## 5 Conclusion

The days of fever were shortened, and the disease progression to respiratory failure in the moderate stage tended to decrease with the addition of KT with SSKKS to conventional treatment. These oral drugs may be an effective treatment choice for fever relief in patients with mild-to-moderate COVID-19. Early application of these Kampo medicines for mild-to-moderate COVID-19 patients may contribute to treating most patients by reducing medical care burden and medical expenses and providing economic and medical benefits.

## Data Availability

The datasets generated and/or analyzed during the current study are available from the corresponding author on reasonable request.

## Supplementary material

SUPPLEMENTARY TABLE S1 Crude drugs included in kakkonto and shosaikotokakikyosekko.

SUPPLEMENTARY TABLE S2 Plant names and part of each ingredient of kakkonto and shosaikotokakikyosekko.

## Abbreviations

CRP, C-reactive protein; CSSE, Center for Systems Science and Engineering; CT, Computed tomography; DPPN, Division of Pharmacognosy, Phytochemistry, and Narcotics; HPLC, High-performance liquid chromatography; IMJEDI, Integrative Management in Japan for Epidemic Disease; JHU, Johns Hopkins University; JP, japanese pharmacopoeia; NRS, Numerical rating scale.
