# Supplementary material for: Multicenter, randomized controlled trial of traditional Japanese medicine, kakkonto with shosaikotokakikyosekko, for mild and moderate coronavirus disease patients
Source: Front Pharmacol. 2022 Nov 9;13:1008946. doi: 10.3389/fphar.2022.1008946 (PMC9682103; doi:10.3389/fphar.2022.1008946)
Supplement: Supplementary file 1 [file Table1.docx]

**Supplementary Table 1. Crude drugs included in kakkonto and shosaikotokakikyosekko**

| **Name of the Kampo medicine** | **Component crude drugs** |
| --- | --- |
| Kakkonto | 4.0 g of JP Pueraria Root, 3.0 g of JP Jujube, 3.0 g of JP Ephedra Herb, 2.0 g of JP Glycyrrhiza, 2.0 g of JP Cinnamon Bark, 2.0 g of JP Peony Root, 2.0 g of JP Ginger |
| Shosaikotokakikyosekko | 10.0 g of JP Gypsum, 7.0 g of JP Bupleurum Root, 5.0 g of JP Pinellia Tuber, 3.0 g of JP Scutellaria Root, 3.0 g of JP Platycodon Root, 3.0 g of JP Jujube, 3.0g of JP Ginseng, 2.0 g of JP Glycyrrhiza, 1.0 g of JP Ginger |

Japanese Pharmacopoeia (JP)
