# Supplementary material for: Multicenter, randomized controlled trial of traditional Japanese medicine, kakkonto with shosaikotokakikyosekko, for mild and moderate coronavirus disease patients
Source: Front Pharmacol. 2022 Nov 9;13:1008946. doi: 10.3389/fphar.2022.1008946 (PMC9682103; doi:10.3389/fphar.2022.1008946)
Supplement: Supplementary file 2 [file Table2.docx]

**Supplementary Table 2. Plant names and part of each ingredient of kakkonto and shosaikotokakikyosekko**

| **Ingredient in English** | **Plant name (Latin)** | **Plant part (Latin)** |
| --- | --- | --- |
| JP Bupleurum Root | *Bupleurum falcatum* Linné (*Umbelliferae*) | *Radix* |
| JP Cinnamon Bark | *Cinnamomum cassia* Blume (*Lauraceae*) | *Cortex* |
| JP Ephedra Herb | *Ephedra sinica* Stapf, *Ephedra intermedia* Schrenk et C. A. Meyer, or *Ephedra equisetina* Bunge (*Ephedraceae*) | *Herba* |
| JP Ginger | Zingiber officinale Roscoe (*Zingiberaceae*) | *Rhizoma* |
| JP Ginseng | *Panax ginseng* C. A. Meyer (*Panax schinseng* Nees) (Araliaceae) | *Radix* |
| JP Glycyrrhiza | *Glycyrrhiza uralensis* Fischer, or *Glycyrrhiza glabra* Linné (*Leguminosae*) | *Radix* |
| JP Gypsum | *Gypsum fibrosum* | *-* |
| JP Jujube | *Zizyphus jujuba* Miller var. *inermis* Rehder (*Rhamnaceae*) | *Fructus* |
| JP Peony Root | *Paeonia lactiflora* Pallas (*Paeoniaceae*) | *Radix* |
| JP Pinellia Tuber | *Pinellia ternata* Breitenbach (*Araceae*) | *Tuber* |
| JP Platycodon Root | *Platycodon grandiflorum* A. De Candolle (*Campanulaceae*) | *Radix* |
| JP Pueraria Root | *Pueraria lobata* Ohwi (*Leguminosae*) | *Radix* |
| JP Scutellaria Root | *Scutellaria baicalensis* Georgi (*Labiatae*) | *Radix* |
